# Physicochemical, nutritional, and flavor attributes of Dangshan Su pear fermented with various lactic acid bacteria

**DOI:** 10.1016/j.fochx.2026.104338

**Published:** 2026-08-27

**Authors:** Jiayu Gu, Qian Zhu, Jingjing Du, Jiagang Guo, Yuhan Wu, Shuo Wang, Jian Jiang, Song Yang

**Affiliations:** a Institute of Agro-products Processing, Anhui Academy of Agricultural Sciences, Hefei, Anhui 230041, China; b Anhui Engineering Laboratory for Functional Microorganisms and Fermented Foods, Anhui Academy of Agricultural Sciences, Hefei, Anhui 230041, China

**Keywords:** Pear, Microbial fermentation, Bioactive compounds, Flavor, Metabolic profiling

## Abstract

This study evaluated eight lactic acid bacteria (LAB) strains for effects on the physicochemical properties, antioxidant activity, and volatile metabolites of Dangshan Su pear. Two selected strains (*Lactiplantibacillus plantarum* L3 and *Limosilactobacillus fermentum* R11) were further characterized for non-volatile metabolite profiles in single and mixed cultures. LAB fermentation significantly enhanced total phenolics and flavonoids, DPPH/ABTS^+^ scavenging, and α-glucosidase/α-amylase inhibition, with L3 showing the greatest improvement. Aroma remodeling was strain-dependent, with L3 boosting fruity esters and R11 improving mouthfeel. Non-targeted metabolomics revealed extensive remodeling, particularly in the co-culture (L3 + R11). Various metabolites (isoleucine, valine, proline, GABA, choline, and dipeptides) with VIP > 1.0 were significantly increased following fermentation, contributing to flavor formation and nutritional enhancement. Notably, GABA increased by 4.62-fold after R11 fermentation. These results demonstrate that L3 and R11 are effective starter cultures for enhancing pear flavor and nutritional value, offering a feasible strategy for the high-value utilization of pear resources.

## Highlights

• Dangshan Su pear was a suitable substrate for lactic acid bacteria.
• LAB fermentation significantly enhanced total phenolic/flavonoid contents.
• LAB fermentation increased antioxidant activity and amylase/glucosidase inhibition.
• LAB fermentation improves the flavor and nutritional value of Dangshan Su pears.
• *L. plantarum* L3 and *L. fermentum* R11 are suitable starter cultures.

## 1 Introduction

China is one of the origins of pears, and *Pyrus bretschneideri* cv. Dangshan Su occupies the largest planting area among domestic pear varieties (Jin et al., 2013). However, as seasonal fruits, fresh consumption alone cannot absorb its large output, necessitating deep processing to address the oversupply (Hou et al., 2022). In Anhui Province, Dangshan County serves as a major processing hub producing juice, canned pears, and paste, yet these products suffer from low added value, outdated technology, and pronounced homogenization (Chen et al., 2025). Therefore, developing high-value products has become a critical technical challenge. Lactic acid bacterial (LAB) fermentation is a well-established strategy to enhance the quality and functionality of fruit products by improving color stability, flavor, and shelf life while generating functional metabolites (Shah et al., 2024). Fermented fruit products represent a promising avenue for functional food innovation (Li et al., 2021; Szutowska, 2020), and distinct LAB strains exhibit unique metabolic traits that determine the final product quality (Gu et al., 2025; Liu et al., 2025). Therefore, screening for strains that produce desirable flavors while avoiding unpleasant or incompatible notes is critical (Rodríguez et al., 2021).

Pear possesses favorable nutritional properties, making it an excellent substrate for LAB fermentation. Various studies have established LAB fermentation as an effective strategy to enhance the nutritional quality, functional properties, and flavor profiles of pear-based products. Jiang et al. (2025) demonstrated that *Lactiplantibacillus plantarum* MPS-PJ241 and *Lacticaseibacillus paracasei* TD-PJ242 adapted well to Dangshan Su pear juice, with viable counts exceeding 12 log CFU/mL post-fermentation. They reduced the total weight and average particle size of stone cells, enhanced antioxidant capacity and shelf-life stability, and altered metabolic profiles via amino acid biosynthetic pathways (Jiang et al., 2025). Similarly, Jin et al. (2024) reported that *Pediococcus pentosaceus* JS91 and JS96 improved the total antioxidant capacity and overall sensory acceptance of Dangshan Su pear juice. Furthermore, the impact of LAB fermentation on flavor profiles has been explored. In particular, Wang et al. (2022) revealed that *Lactobacillus* fermentation enhanced the flavor profile of Huangguan pear juice by promoting desirable volatiles (alcohols, esters, acids, and terpenoids) while reducing aldehydes and ketones.

Nevertheless, systematic comparisons among different LAB strains for fermenting Dangshan Su pear products remain limited. While previous research has confirmed the feasibility of LAB fermentation for pear juice, these studies have largely focused on single or a few strains. Crucially, the eight selected LAB strains in this study have not been previously evaluated in pear fermentation. Their specific metabolic behaviors and suitability for this specific fruit matrix remain entirely unexplored. Furthermore, although metabolomics has been employed in some pear juice studies, comprehensive strain-specific metabolic profiling in Dangshan Su pear has not been reported. Therefore, this study aimed to systematically evaluate eight LAB strains for the fermentation of Dangshan Su pear. Beyond conventional physicochemical and sensory analyses, we performed volatile and untargeted metabolic profiling to globally map the strain-specific metabolic regulatory networks. This integrated approach enables us not only to identify suitable strains for producing pear products, but also to elucidate the differential metabolic mechanisms underlying their distinct quality attributes. This bridges the gap between strain-specific microbial metabolism and the final quality attributes of fermented pears.

## 2 Materials and methods

### 2.1 Materials and chemicals

Dangshan Su pears were purchased from Dangshan (Anhui, China). De Man, Rogosa, and Sharpe (MRS) agar media were obtained from Sinopharm Chemical Reagents Co., Ltd. (Shanghai, China). DPPH and ABTS were purchased from Yuanye Company Ltd. (Wuhan, China). α-glucosidase and α-amylase was obtained from Aladdin (Shanghai, China). Organic acid standards were obtained from Beijing Wokai Biotechnology Co. Ltd.

### 2.2 Activation and culture of the strains for fermentation

The strains used for Dangshan Su pear fermentation were selected from our previous isolates strains originated from diverse fruits and vegetables collected in Anhui Province. The detailed strain information is presented in Table S1. Strain selection was based on preliminary screening experiments. Specifically, all candidate strains were first evaluated for safety profiles (specifically, hemolytic activity). Subsequently, the strains that exhibited negative hemolytic activity were further assessed for growth performance in the Dangshan Su pear matrix. Only those capable of robust growth in the pear substrate were selected. Although these strains have not been previously reported in pear fermentation, their 16S rRNA gene sequences have been deposited in the NCBI database under accession numbers PZ770652–PZ770659 (https://www.ncbi.nlm.nih.gov/genbank/) to ensure traceability. The strains were maintained at −80 °C in MRS broth containing 30% (*v*/v) glycerol. The frozen strains were incubated at 37 °C for 24 h and then inoculated twice into liquid MRS medium. The activated strains were washed twice with PBS and inoculated into Dangshan Su pear for fermentation.

### 2.3 Preparation of Dangshan Su pear cans

Fresh Dangshan Su pears were washed with running tap water, peeled, and sliced into uniformly sized pieces without additional sanitization. The slices were then mixed with a 15% (*w*/w) sucrose aqueous solution (prepared with distilled water) at a 1:1 (*w*/w) ratio, filled into glass jars, and pasteurized at 90 °C for 20 min prior to inoculation with lactic acid bacteria. The activated strains were harvested by centrifugation (6000×*g*, 10 min, 4 °C), washed twice with sterile PBS (0.01 M, pH 7.2), and resuspended in PBS to a final concentration of approximately 1.0 × 10^7^ CFU/mL, as determined by plate counting on MRS agar. The inoculum was added at 1% (*v*/*w*). Fermentation was conducted in sealed 300 mL glass jars under static conditions at 37 ± 1 °C. Samples were collected at 0, 4, 8, 12, 16, and 20 h for pH monitoring. The 20 h fermentation time was chosen based on preliminary experiments showing that all strains reached stationary phase (viable counts >10^9^ CFU/mL) with stable pH by this point. All fermentations were performed in three independent biological replicates, each with two technical replicates. After fermentation, samples were homogenized and stored at −80 °C until analysis. Control samples were inoculated with an equal volume of sterile PBS and incubated under the same conditions.

### 2.4 Physicochemical properties of Dangshan Su pear before and after fermentation

We next examined the pH, soluble solids, total acid, and total sugar content of Dangshan Su pears before and after fermentation. All measurements were performed in triplicate for each independent biological replicate (*n* = 3). The pH and soluble solids content were measured with a pH meter (PHS-3C, Shanghai Leici, China) and a refractometer (PAL-1, Atago, Tokyo, Japan), respectively. The total acid content was determined by acid-base indicator titration. Briefly, the sample solution (15 mL) was titrated with 0.1 mol/L NaOH standard solution for 30 s until it exhibited a slight red color without further change. The volume of NaOH consumed was recorded to calculate the total acid content of the sample, and the results are expressed as lactic acid content. The total sugar content was determined using the phenol‑sulfuric acid method (Shi et al., 2023). Briefly, d-glucose standard (dried at 90 °C) was prepared at 0.1 mg/mL. Aliquots (0, 0.2, 0.4, 0.6, 0.8, 1.0 mL) were adjusted to 1.0 mL with distilled water, mixed with 0.5 mL of 6% phenol and 2.5 mL of concentrated H_2_SO_4_, then incubated at room temperature for 30 min. Absorbance was measured at 490 nm to construct a calibration curve, yielding the standard curve equation y = 4.1116x − 0.0048 (R^2^ = 0.9992). Diluted samples were analyzed similarly, and results were expressed as g glucose equivalents per 100 mL.

### 2.5 Determination of organic acids of Dangshan Su pear before and after fermentation

The contents of tartaric acid, oxalic acid, citric acid, malic acid, lactic acid, and acetic acid were determined using high-performance liquid chromatography (HPLC, LC-20 A, Shimadzu, Kyoto, Japan). All measurements were performed in triplicate independent biological replicates (*n* = 3). Fermented and unfermented Dangshan Su pear samples were homogenized. A 10 mL sample was centrifuged at 10,000 rpm for 10 min, and the supernatant was filtered twice through a 0.22 μm filter membrane. The separation was performed on an XBridge C18 analytical column (4.6 mm × 250 mm, 5 μm, Waters, Milford, MA, USA) with a mobile phase consisting of 0.1% phosphoric acid and methanol (97:3 *v*/v) at 30 °C, with an injection volume of 10 μL. The eluted compounds were detected at 210 nm using a photodiode array detector (SPD-M20A, Shimadzu, Kyoto, Japan). Individual organic acids were identified by comparison of their retention times with those of authentic standards (Table S2). Organic acids were quantified by the external standard method. Individual stock solutions (1.0 mg/mL) of tartaric, oxalic, citric, malic, lactic, and acetic acids (≥98% purity, Sigma-Aldrich) were prepared in ultrapure water and serially diluted to six concentration levels (5–200 μg/mL). Calibration curves (peak area vs. concentration) were constructed for each acid, all with R^2^ > 0.99. Sample concentrations were calculated from the corresponding curves and expressed as mg/L (Table S2).

### 2.6 Determination of total phenol and total flavonoid contents

The total polyphenol and flavonoid contents in the Dangshan Su pears during fermentation were determined as previously described (Wu et al., 2021) and assayed using the Folin–Ciocalteu method and the aluminum chloride (AlCl_3_) colorimetric method, respectively. All measurements were performed in triplicate for each independent biological replicate (*n* = 3). Fermented and unfermented pear homogenates were centrifuged at 10,000×*g* for 10 min at 4 °C, and the supernatant was collected for further analysis. Briefly, gallic acid standard was prepared at 100 μg/mL and diluted to seven concentrations (0–10 μg/mL). Each standard or appropriately diluted sample was mixed with 2.0 mL Folin–Ciocalteu reagent and 3.0 mL 15% Na₂CO₃, incubated for 30 min, and measured at 760 nm, yielding the calibration curve y = 0.149× + 0.0219 (R^2^ = 0.9975). Rutin standard (100 μg/mL in anhydrous ethanol) was diluted to five levels (0, 4, 6, 10, and 12 μg/mL), mixed with 0.3 mL of 5% NaNO₂ for 6 min, followed by 0.3 mL of 10% Al(NO₃)₃ for 6 min, and 2.0 mL of 4% NaOH. The mixture was then made up to 10 mL with distilled water, incubated for 15 min, and measured at 510 nm, yielding y = 0.0116x + 0.00007 (R^2^ = 0.9999). Results were expressed as mg gallic acid equivalents per liter (mg GAE/L) and mg rutin equivalents per liter (mg RE/L), respectively.

### 2.7 Determination of antioxidant and enzyme inhibitory activities

The ABTS^+^ and DPPH scavenging abilities of Dangshan Su pears were detected and calculated according to Shi et al. (2023) with modifications. Briefly, 100 μL of sample was mixed with 100 μL of DPPH working solution (120 μg/mL in anhydrous ethanol) and kept in the dark for 30 min, with absorbance measured at 517 nm. 40 μL of sample was mixed with 4.0 mL of ABTS^+^ working solution (diluted to an absorbance of 0.70 ± 0.02 at 734 nm), incubated in the dark for 2 min, and the absorbance was measured at 734 nm.

The α-amylase and α-glucosidase inhibitory activities of Dangshan Su pears were determined using the method of Shi et al. (2023). 30 μL of PBS (0.1 M, pH 6.8), 20 μL of sample, and 50 μL of soluble starch (1%) were mixed in a 96-well plate and incubated at 37 °C for 5 min, followed by the addition of 20 μL of α-amylase solution (5 μg/mL) and further incubation at 37 °C for 15 min. The reaction was terminated by adding 50 μL of HCl (1 M), and then 50 μL of iodine solution (0.01 M) was added for color development. The absorbance was measured at 650 nm. 100 μL of PBS (0.1 M, pH 6.8), 50 μL of sample, and 20 μL of α-glucosidase solution (20 U/mL in PBS) were incubated at 37 °C for 5 min, followed by the addition of 20 μL of p-NPG solution (5 mmol/L) and further incubation at 37 °C for 30 min. The reaction was terminated by adding 50 μL of Na₂CO₃ (0.2 M), and the absorbance was measured at 405 nm. All measurements were performed in three independent biological replicates, each with three technical replicates.

### 2.8 Determination of volatile compounds

Volatile compounds in unfermented and fermented Dangshan Su pears were determined using headspace solid-phase microextraction gas chromatography–mass spectrometry (Thermo Fisher, CA, USA), equipped with a TR-5MS capillary column (30 m × 0.25 mm × 0.25 μm). 5 g of homogenized Dangshan Su pear sample was placed in a sampling vial containing 1 g of NaCl, with deuterated chlorobenzene as an internal standard. The vials were equilibrated in a water bath at 60 °C for 10 min, then the SPME fiber (50/30 μm CAR/PDMS/DVB) was exposed to the headspace for 30 min, and immediately desorbed in the GC–MS injection port at 250 °C for 5 min. The GC–MS analysis was carried out with modifications based on Shi et al. (2023). The GC injector temperature was set at 200 °C, with helium as the carrier gas at a flow rate of 0.9 mL/min under splitless injection mode and a solvent delay of 5 min. The oven temperature program began at 45 °C held for 1 min, then increased to 100 °C at 5 °C/min and held for 3 min, and finally raised to 250 °C at 6 °C/min with a final hold of 8 min. The mass spectrometer was operated in electron impact ionization mode with the ion source temperature at 200 °C, an electron energy of 70 eV, and a mass scan range from 35 to 500 *m*/*z*. Volatile compounds were identified by comparing their mass spectra with those in the NIST (National Institute of Standards and Technology) mass spectral library (version 2.0), and their retention indices were calculated using a series of n-alkanes (C8–C40) and compared with literature values. All experiments were performed in triplicate (*n* = 3).

### 2.9 Untargeted profiling of non-volatile compounds

Non-volatile compounds in unfermented and fermented Dangshan Su pears (*n* = 5) were analyzed by ultra-high performance liquid chromatography coupled with quadrupole time-of-flight mass spectrometry (UPLC-QTOF-MS, Thermo Fisher Scientific, MA, USA) according to Xu et al. (2020) with modifications. Samples were centrifuged (8000×*g*, 10 min), and the supernatants were extracted with 80% methanol under ultrasonication (40 min, 4 °C, 80 kHz). After another centrifugation (12,000×*g*, 10 min), the supernatants were lyophilized and redissolved in 80% methanol for UPLC-QTOF-MS analysis. Separation was conducted on a Waters Acquity UPLC HSS T3 column (2.1 mm × 100 mm, 1.8 μm) at 35 °C. Mass detection utilized a heated electrospray ionization source in both positive and negative modes. The mobile phases were 0.1% formic acid in water (A) and methanol (B) for positive mode, and water containing 5 mM ammonium formate (A) and methanol (B) for negative mode, at 0.3 mL/min. The gradient program was: 0–1.0 min (2% B), 1.0–10 min (2–98% B), 10–12 min (98% B), and 12–15 min (2% B). ESI voltages were 3.8 kV (positive) and 2.8 kV (negative). MS1 scanning covered *m*/*z* 50–1070 at 70,000 resolution, with an AGC target of 1 × 10^6^ and an injection time of 100 ms. MS2 was performed at 17,500 resolution with collision energies of 20, 40, and 60 eV, an AGC target of 5 × 10^4^, an injection time of 50 ms, a dynamic exclusion of 10 s, and fragmentation of the top 7 ions.

### 2.10 Sensory evaluation

Sensory evaluation was performed using quantitative descriptive analysis (QDA) by a panel of 12 trained assessors (six males and six females, aged 22–45 years) recruited from faculty and graduate students majoring in Food Science. Panelists were selected based on the following inclusion criteria: no known allergies to pear or other fruits, no smoking history, normal olfactory and gustatory function, and availability for all training and evaluation sessions. All panelists had prior sensory experience and completed two 2-h training sessions prior to formal evaluation. Each sample (approx. 20 g) was placed in a coded, odor-free cup, served at 25 ± 2 °C in a randomized order, with water and unsalted crackers provided for palate cleansing. Panelists evaluated each sample based on five attributes: color, acidity, sweetness, aroma, and taste, each scored on a 20-point scale. The scoring criteria for each attribute are detailed in Table S3. The total sensory score was calculated as the sum of the five attribute scores (maximum 100 points). Evaluations were carried out in a dedicated tasting room with individual booths under standard daylight conditions at 22 ± 2 °C, and panelists were blinded to sample identities. The reproducibility of the sensory panel was assessed by calculating the coefficient of variation (CV) for each attribute across the three replicate evaluations, with an acceptable CV threshold of <15%.

This sensory evaluation was approved by the Science and Technology Ethics Committee of the Institute of Agro-Products Processing Research, Anhui Academy of Agricultural Sciences (Approval No. AAAS-JGS2026201). The study was conducted in accordance with the ethical principles of the Declaration of Helsinki and its subsequent amendments. All panelists were informed of the study purpose, their right to withdraw at any time without consequence, and the confidentiality of their responses. Written informed consent was obtained from all participants prior to the evaluation. All samples evaluated were confirmed safe for consumption. Appropriate protocols were utilized to protect the rights and privacy of all participants; personal data were anonymized and are not disclosed in this manuscript.

### 2.11 Statistical analysis

The results are presented as mean ± standard error. Normality and homogeneity of variances were assessed by Shapiro–Wilk and Levene's tests, respectively. Differences between groups were assessed using analysis of variance (ANOVA), followed by Fisher's least significant difference (LSD) post hoc test for multiple comparisons. Different lowercase letters (a, b, c, d, e, f, and g) indicate significant differences among the groups (*p < 0.05*). Graphics and analyses were performed using Origin 2024 (OriginLab, USA) and SPSS 16.0. Pearson's correlation analysis was conducted based on the mean values using Origin 2024. The correlation heatmap was visualized to show the pairwise correlation coefficients (r). A Pearson's correlation network of volatiles and sensory attributes was constructed via MetaboAnalyst 6.0 (http://www.metaboanalyst.ca), retaining correlations with |r| > 0.5 and *p < 0.05* to identify key biomarkers.

The raw UPLC-QTOF-MS data were preprocessed and normalized using the Pareto scaling method. Principal component analysis (PCA), partial least squares discriminant analysis (PLS-DA), and orthogonal partial least squares discriminant analysis (OPLS-DA) were performed using the online platform of MetaboAnalyst 6.0. The robustness of the PLS-DA models was validated by 7-fold cross-validation and permutation tests (*n* = 200) to prevent overfitting. Differential metabolites were screened based on a multi-criteria strategy incorporating the Variable Importance in Projection (VIP) score derived from the PLS-DA model, the magnitude of abundance changes, and statistical significance. Specifically, metabolites with a VIP score > 1.0, |log_2_(fold change)| > 1, and *p < 0.05* in the paired Student's *t*-test were considered as candidates for differential regulation. The Benjamini–Hochberg procedure was applied to control the false discovery rate (FDR), and metabolites with q < 0.05 were retained as significantly regulated.

## 3 Results and discussion

### 3.1 Post-fermentation physicochemical properties of Dangshan Su pear

In this study, previously isolated LAB were used to ferment Dangshan Su pear pieces. The pH significantly decreased from 5.46 ± 0.02 to a minimum of 3.91 ± 0.01 in R11-fermented samples after 20 h of fermentation (*p < 0.05*, Table 1). This decrease is attributed to the accumulation of organic acids produced via glycolysis during LAB carbohydrate metabolism. The total acid content of the unfermented Dangshan Su pear pieces was 0.17 ± 0.02 g/L. All eight strains significantly increased their total acid content after fermentation, with R11 showing the largest increase at 5.84 ± 0.03 g/L, likely due to its higher carbohydrate utilization efficiency and homofermentative metabolic activity.

**Table 1:** Physicochemical properties of Dangshan Su pear after fermentation.

Groups	pH	Soluble solids (°Brix)	Total acid (g/L)	Total sugar (g/L)
Control	5.46 ± 0.02^a^	12.77 ± 0.15^a^	0.17 ± 0.02^a^	131.32 ± 1.21^a^
L3	4.26 ± 0.02^b^	12.40 ± 0.10^b^	1.25 ± 0.01^b^	119.16 ± 1.47^b^
R11	3.91 ± 0.01^h^	10.13 ± 0.15^f^	5.84 ± 0.03^h^	98.07 ± 0.25^e^
L11	4.18 ± 0.01^c^	12.23 ± 0.15^bc^	1.89 ± 0.04^c^	113.24 ± 2.63^c^
R7	4.09 ± 0.01^e^	11.96 ± 0.06^c^	2.15 ± 0.02^d^	107.68 ± 1.17^d^
R9	4.09 ± 0.01^e^	11.23 ± 0.15^d^	2.26 ± 0.01^d^	107.61 ± 3.19^d^
R6	4.04 ± 0.01^f^	10.27 ± 0.06^ef^	2.59 ± 0.02^e^	101.63 ± 1.32^e^
R13	4.09 ± 0.01^e^	11.10 ± 0.10^d^	2.52 ± 0.02^e^	102.24 ± 0.76^e^
F3	4.15 ± 0.01^d^	12.00 ± 0.10^c^	1.91 ± 0.02^c^	111.24 ± 4.36^d^

Meanwhile, the LAB strains significantly reduced the total sugar and soluble solids content through their metabolic consumption. R11 fermentation resulted in the lowest total sugar (from 131.32 ± 1.21 to 98.07 ± 0.25 g/L) and soluble solids (10.13 ± 0.15°Brix). Conversely, L3 showed lower sugar utilization, with a remaining total sugar of 119.16 ± 1.47 g/L, suggesting strain-specific differences in metabolic capacity. Therefore, all fermented Dangshan Su pear samples consistently exhibited lower pH, soluble solids, and total sugar values, but higher total acid content compared to the unfermented control. These findings are consistent with previous reports on LAB-fermented pear and other fruit juices, which similarly observed decreased pH and increased acidity alongside reduced soluble solids and sugars due to microbial carbohydrate metabolism (Jin et al., 2024; Wang et al., 2022). The notable inter-strain variation in acidification and sugar consumption highlights the importance of selecting specific LAB strains to achieve targeted fermentation profiles in fruit products.

### 3.2 Organic acid contents of Dangshan Su pear

**Fig. 1:**
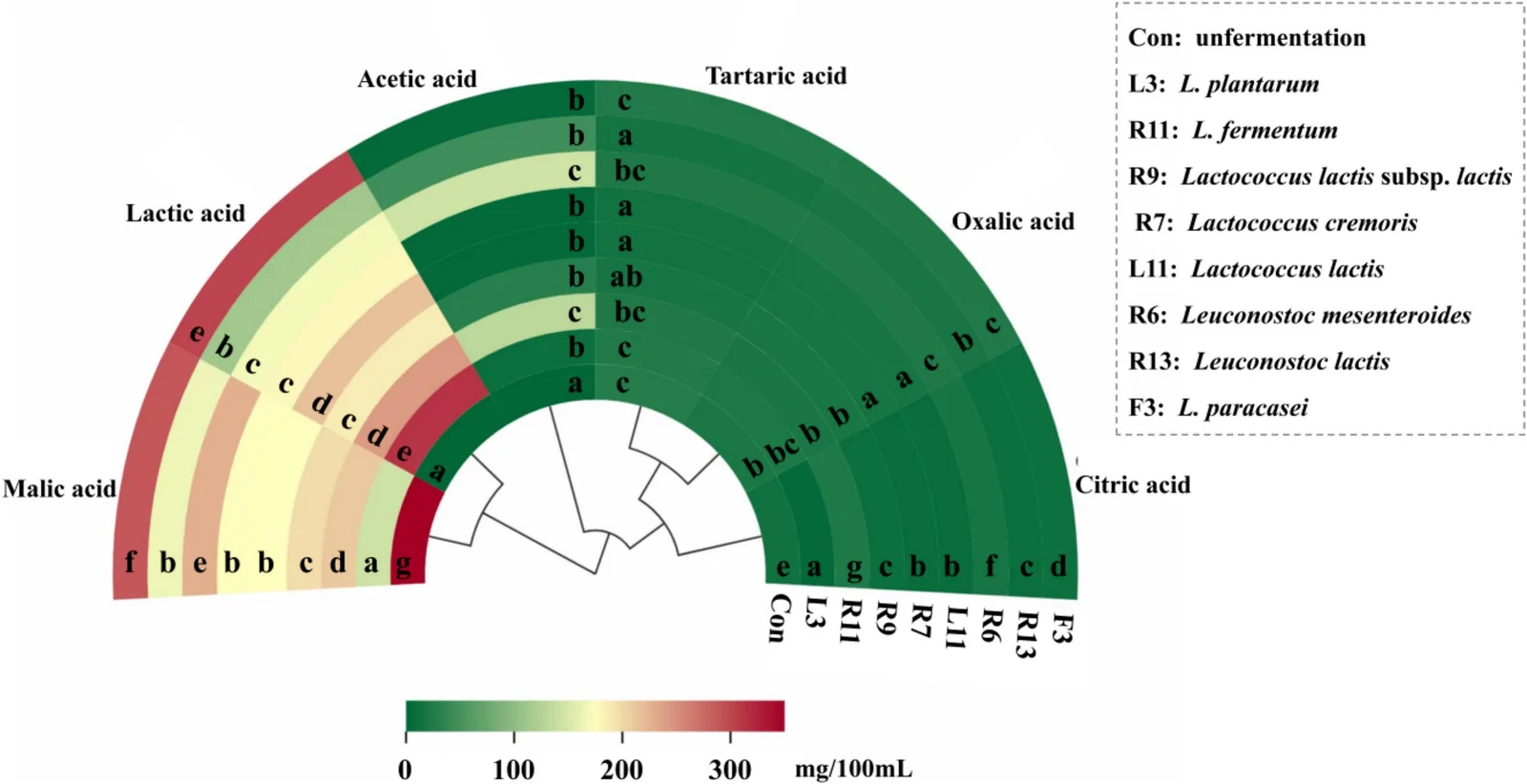
Organic acid profiles of Dangshan Su pear fermented by LAB strains (Con: unfermented control, L3: *L. plantarum*, R11: *L. fermentum*, L11: *Lactococcus lactis*, R7: *Lactococcus cremoris*, R9: *Lactococcus lactis* subsp. *lactis*, R6: *Leuconostoc mesenteroides*, R13: *Leuconostoc lactis*, F3: *L. paracasei*). Different lowercase letters (a–g) indicate significant differences among groups (one-way ANOVA followed by LSD test, *p < 0.05*).

Organic acids significantly influence the taste, flavor, and nutritional quality of fermented pears. Tartaric acid, oxalic acid, citric acid, malic acid, lactic acid, and acetic acid contents in Dangshan Su pears were investigated before and after fermentation (Fig. 1). In unfermented Dangshan Su pears, malic acid was the predominant organic acid, reaching 318.97 ± 0.84 mg/100 mL. Tartaric acid, oxalic acid, and citric acid were present in lower amounts, whereas lactic and acetic acids were not detected. Following LAB fermentation, the contents of tartaric acid, oxalic acid, citric acid, and malic acid decreased remarkably, with malic acid showing the greatest reduction, particularly in samples fermented by *L. plantarum* L3 (130.22 ± 1.10 mg/100 mL). This decrease is attributed to the conversion of malic acid to lactic acid and CO_2_ via malolactic fermentation catalyzed by malolactic enzyme, since malic acid serves as an important secondary carbon source for LAB proliferation (Ji et al., 2023). These trends are consistent with previous findings in pear juice fermentation, where LAB fermentation similarly reduced malic, oxalic, citric, and tartaric acids (Wang et al., 2022).

Conversely, lactic acid content increased substantially after LAB fermentation, becoming the major organic acid in all fermented samples. *L. plantarum* L3 exhibited the highest lactic acid production (240.85 ± 8.61 mg/100 mL) with relatively low acetic acid content, whereas *L. fermentum* R11 produced high levels of both lactic acid (220.37 ± 5.54 mg/100 mL) and acetic acid (131.02 ± 0.74 mg/100 mL). The production of acetic acid is attributable to the heterofermentative metabolic pathway of certain LAB strains, which metabolize hexoses via the phosphoketolase pathway, yielding equimolar amounts of lactic acid, acetic acid (or ethanol), and CO₂ (Gänzle, 2015). In contrast, homofermentative strains such as *L. plantarum* L3 primarily convert sugars to lactic acid via glycolysis, resulting in lower acetic acid production. *L. lactis* R9, R13, and L. *paracasei* F3 also showed strong capacity for lactic and acetic acid production. Notably, the lactic acid yield of *L. plantarum* L3 was substantially higher than the levels (e.g., approximately 50–70 mg/100 mL) reported in pear juice fermented with other *Lactobacillus* strains (Wang et al., 2022), highlighting its acid-producing potential for pear fermentation.

### 3.3 Total phenol and flavonoid contents of Dangshan Su pear

**Fig. 2:**
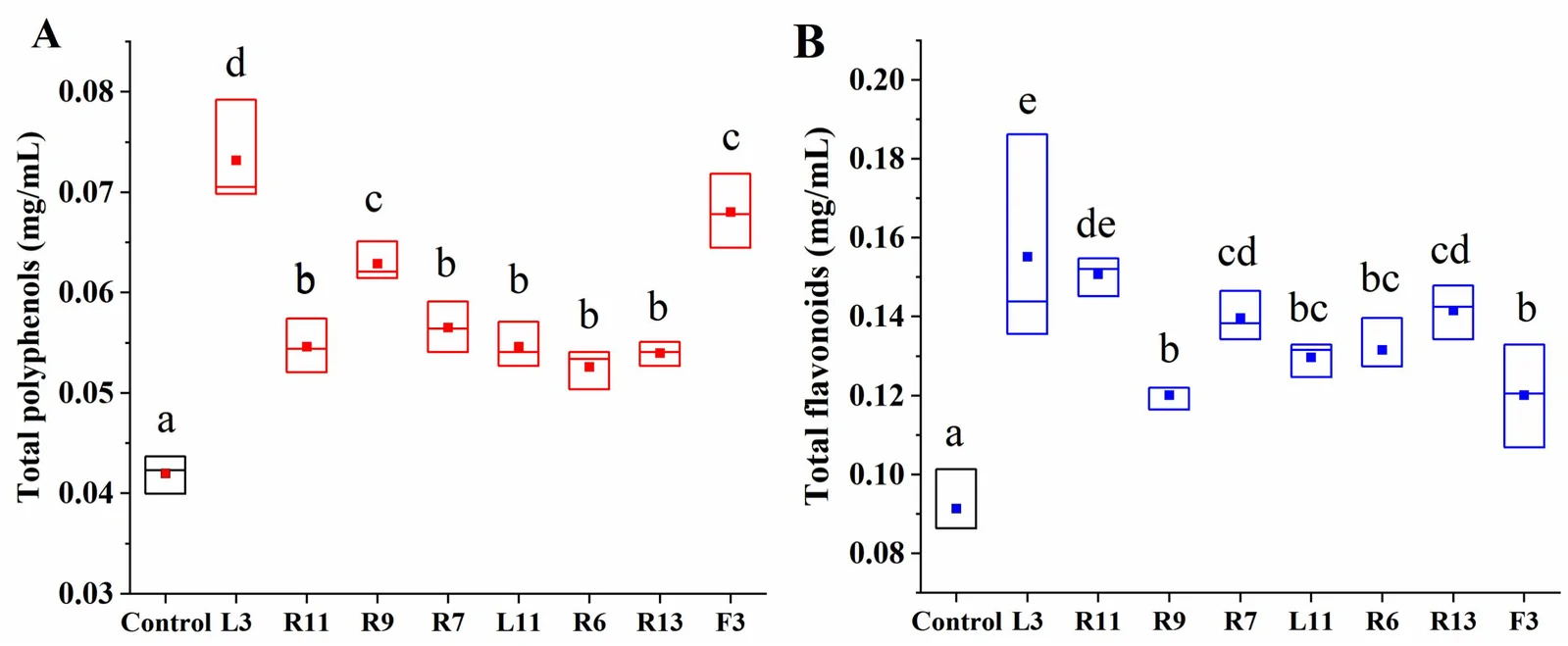
Total polyphenol (A) and flavonoid (B) contents of Dangshan Su pears following different LAB fermentation. (Con: unfermented control, L3: *L. plantarum*, R11: *L. fermentum*, L11: *Lactococcus lactis*, R7: *Lactococcus cremoris*, R9: *Lactococcus lactis* subsp. *lactis*, R6: *Leuconostoc mesenteroides*, R13: *Leuconostoc lactis*, F3: *L. paracasei*). Different lowercase letters (a–e) indicate significant differences among groups (one-way ANOVA followed by LSD test, *p < 0.05*).

Phenolic compounds and flavonoids are important bioactive substances in fruits and vegetables that influence nutritional and sensory qualities. The effects of different LAB fermentations on the total phenolic and flavonoid contents of Dangshan Su pear pieces are illustrated in Fig. 2. The total phenolic content in unfermented Dangshan Su pears was 0.04 ± 0.002 mg/mL. All strains significantly increased the total phenolic content. *L. plantarum* L3 showed the highest increase, reaching 0.073 ± 0.02 mg/mL, followed by *L. fermentum* R11 at 0.054 ± 0.01 mg/mL. The initial total flavonoid content was 0.09 ± 0.01 mg/mL, which was significantly increased after fermentation. *L. plantarum* L3 fermentation resulted in the greatest increase in total flavonoids, with a 72.50% rise compared to the pre-fermentation level.

LAB fermentation significantly enhances the total phenolic and flavonoid content of various fruit matrices. Consistent with our findings, Xie et al. (2025) demonstrated that mixed-strain fermentation of apple puree resulted in 62.78% and 29.48% increases in total phenolics and flavonoids, respectively. The increases in total phenolics and flavonoids can be attributed to the β-glucosidase activity of LAB, which hydrolyzes glycosidic bonds to release free aglycones with enhanced antioxidant activity (Rodríguez et al., 2021). This deglycosylation process enhances both the measurable phenolic content and the bioavailability of these compounds, as free aglycones generally exhibit stronger antioxidant activity than their glycosylated counterparts (Isas et al., 2020). However, different LAB strains secrete various enzymes during fermentation and exert varying effects on phenolics and flavonoids. *L. plantarum* increases the total phenolic content to a greater extent than other LAB, because it possesses unique genes capable of synthesizing phenol decarboxylases and tannases, demonstrating strong enzymatic activity in polyphenol metabolism (Kwaw et al., 2018; Piekarska-Radzik & Klewicka, 2021).

### 3.4 Post-fermentation antioxidant and α-glucosidase/α-amylase inhibition activities of Dangshan Su pear

**Fig. 3:**
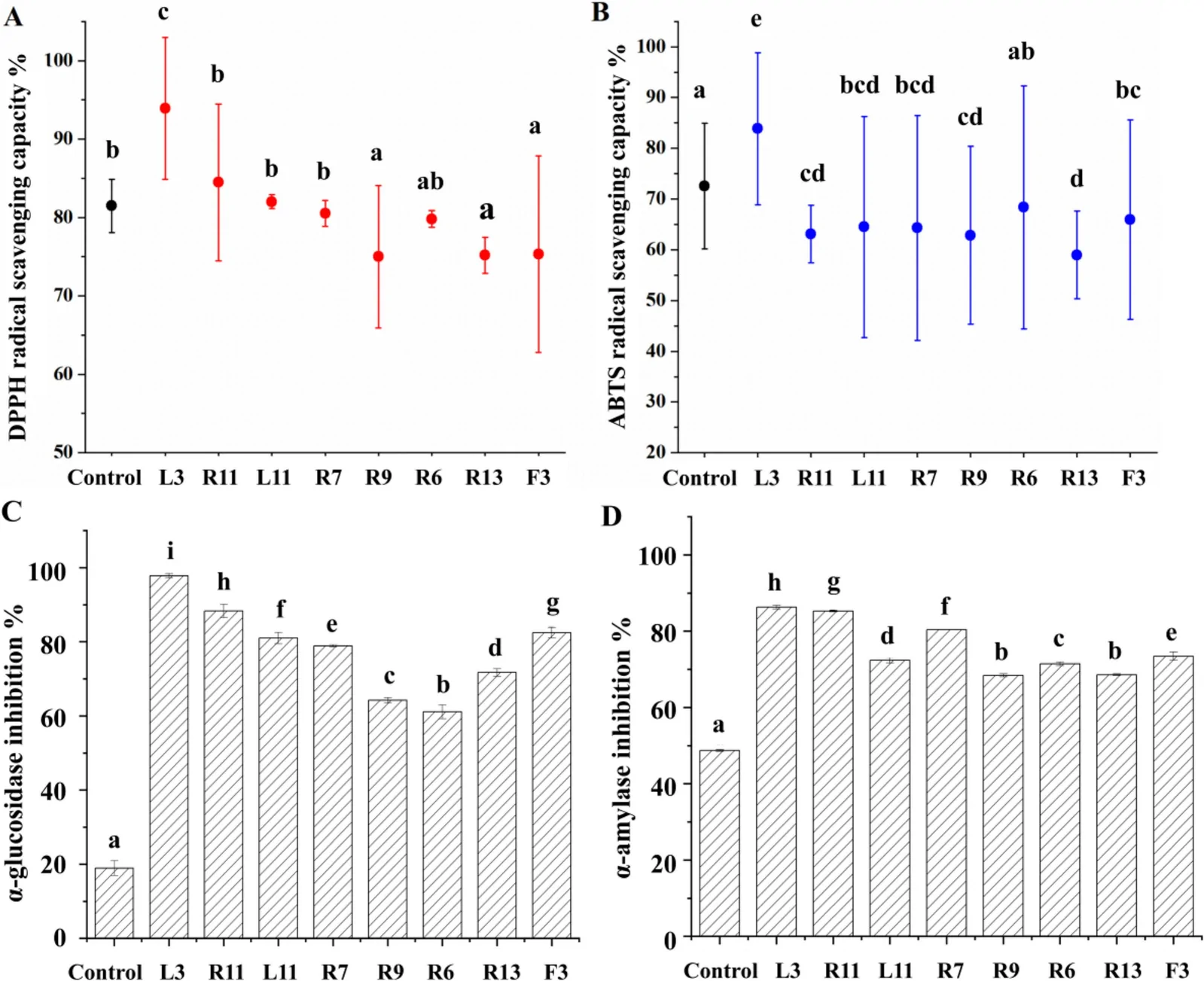
ABTS^+^ (A) and DPPH (B) scavenging activities, as well as α-glucosidase (C) and α-amylase (D) inhibition activities of Dangshan Su pear following different LAB fermentation. (Con: unfermented control, L3: *L. plantarum*, R11: *L. fermentum*, L11: *Lactococcus lactis*, R7: *Lactococcus cremoris*, R9: *Lactococcus lactis* subsp. *lactis*, R6: *Leuconostoc mesenteroides*, R13: *Leuconostoc lactis*, F3: *L. paracasei*). Different lowercase letters (a–g) indicate significant differences among groups (one-way ANOVA followed by LSD test, *p < 0.05*).

We next examined the DPPH and ABTS^+^ radical scavenging capacities of Dangshan Su pears fermented with different LAB strains (Fig. 3). Dangshan Su pears have a higher antioxidant capacity than other cultivars (Xia et al., 2012). In this study, the DPPH and ABTS^+^ radical-scavenging capacities of canned Dangshan Su pear homogenate were 81.50% and 72.58%, respectively. Furthermore, the antioxidant capacity of Dangshan Su pears was significantly enhanced following L3, R11, L11, R7, and F3 fermentations. *L. plantarum* L3 fermentation yielded the highest DPPH and ABTS^+^ radical-scavenging capacities, reaching 93.93% and 83.87%, respectively.

To elucidate the potential mechanism underlying the enhanced biological activities, a Pearson correlation analysis was conducted to investigate the relationships among organic acids, total phenol/flavonoid contents, and the observed functional activities (Fig. S1). Total polyphenols displayed strong positive correlations with both DPPH (*r* = 0.84, *p < 0.01*) and ABTS^+^ (*r* = 0.72, *p < 0.01*). Similarly, total flavonoids were strongly linked to ABTS^+^ scavenging activity (*r* = 0.64, *p < 0.01*). These statistical findings are in agreement with the quantitative data, where strains with higher phenolic content (L3, which showed a distinct increase compared to the control) exhibited superior radical scavenging capacities. The strong positive correlations shown in our heatmap are consistent with previous studies demonstrating that total phenolics and flavonoids largely account for the antioxidant capacity of pear cultivars. Notably, flavonoid content was identified as a critical determinant in Dangshan Su pears (Xia et al., 2012; Salta et al., 2010).

Fermentation by specific LAB can produce bioactive compounds that inhibit α-amylase or α-glucosidase. The α-glucosidase and α-amylase inhibitory activities of Dangshan Su pear were significantly improved following different LAB fermentation. L3 fermentation showed the most significant increase in α-glucosidase inhibition, from 18.93% to 97.80% (an increase by 78.87%), followed by an increase to 88.31% through R11 fermentation. LAB also significantly enhanced the α-amylase inhibitory capacity of Dangshan Su pear by 19.87%–37.77%. *L. plantarum* L3 and *L. fermentum* R11 exerted greater inhibitory effects on α-amylase activity in Dangshan Su pears. Consistently, Wang and Li, 2022 reported that *L. plantarum* exhibited the strongest hypoglycemic activity among nine LAB strains.

The correlation matrix revealed that lactic acid serves as a central functional hub. It exhibited a remarkably strong positive correlation with α-glucosidase inhibition (*r* = 0.85, *p < 0.01)*. In contrast, malic acid showed a significant negative correlation with α-glucosidase (*r* = −0.74, *p < 0.01*). In accordance with this statistical evidence, the accumulation of lactic acid in L3 increased dramatically compared with the control, coinciding with its significantly elevated α-glucosidase inhibition. Beyond the role of organic acids, previous studies have proposed that free aglycones released by β-glucosidase exert stronger enzyme inhibition than their glycosylated forms, while bioactive peptides and organic acids generated during fermentation may contribute through synergistic interactions (Wang and Li, 2022).

### 3.5 Volatile compounds of Dangshan Su pear after fermentation

**Fig. 4:**
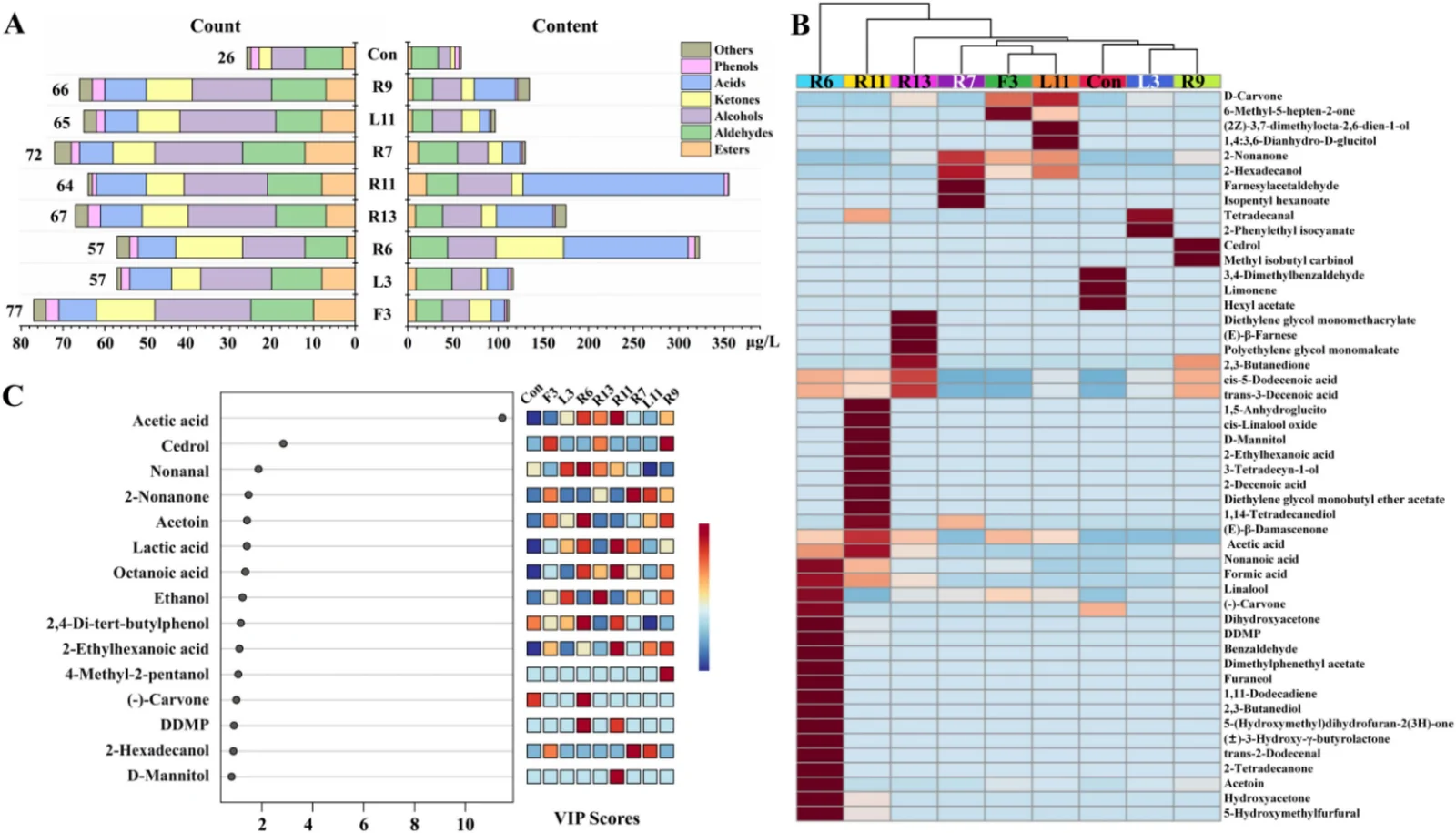
Volatile compounds profiling of Dangshan Su pears following LAB fermentation (Con, L3, R11, L11, R7, R9, R6, R13, and F3). (A) The counts and contents of volatile compounds. (B) Heatmap of differential volatiles (top 50, *p < 0.05*, *n* = 3). (C) VIP scores derived from the PLS-DA model. Compounds with VIP > 1 were considered key discriminants.

To investigate the effects of LAB fermentations on the aroma traits, volatile compounds were analyzed by HS-SPME-GC–MS (Fig. 4). A total of 170 volatile compounds were identified, including 27 esters, 28 aldehydes, 31 ketones, 54 alcohols, and 15 acids. The volatile compounds in unfermented Dangshan Su pears were primarily alcohols, ketones, and aldehydes, with no detectable acids, providing a pleasant fruity flavor. Nonanal, decanal, and hexanal were the predominant flavor compounds, at concentrations of 9.37, 8.77, and 4.65 μg/L, respectively. Decanal and hexanal have a fresh, oily, and fruity aroma, while nonanal, typically detected in apples and pears, provides pears with a strong, greasy, rose, and sweet orange flavor (Qin et al., 2012). Moreover, higher contents of hexyl acetate contributed pear-like notes, whereas ethyl butyrate contributed pineapple-like notes (Melgarejo et al., 2011).

Fermentation with LAB strains significantly increased the diversity and abundance of volatile compounds in Dangshan Su pears. F3 yielded the highest number of volatile compound species, whereas R11 and R6 yielded the highest total contents, at 355.40 and 322.88 μg/L, respectively. This increase was largely driven by the elevated contents of acids, ketones, and aldehydes. Based on variable importance in projection (VIP) scores, the key volatile flavor substances were identified post-fermentation. LAB strains differentially modulated the production of organic acids, lipid-derived aldehydes/ketones, and terpenoid compounds during Dangshan Su pear fermentation, with acetic acid, cedrol, nonanal, and 2-nonanone being the most critical discriminants. Acetic acid is a key metabolite in heterofermentative *Lactobacillus* metabolism via the phosphoketolase pathway. Similarly, lactic acid is an important indicator of homofermentative or mixed-acid fermentation pathways.

**Fig. 5:**
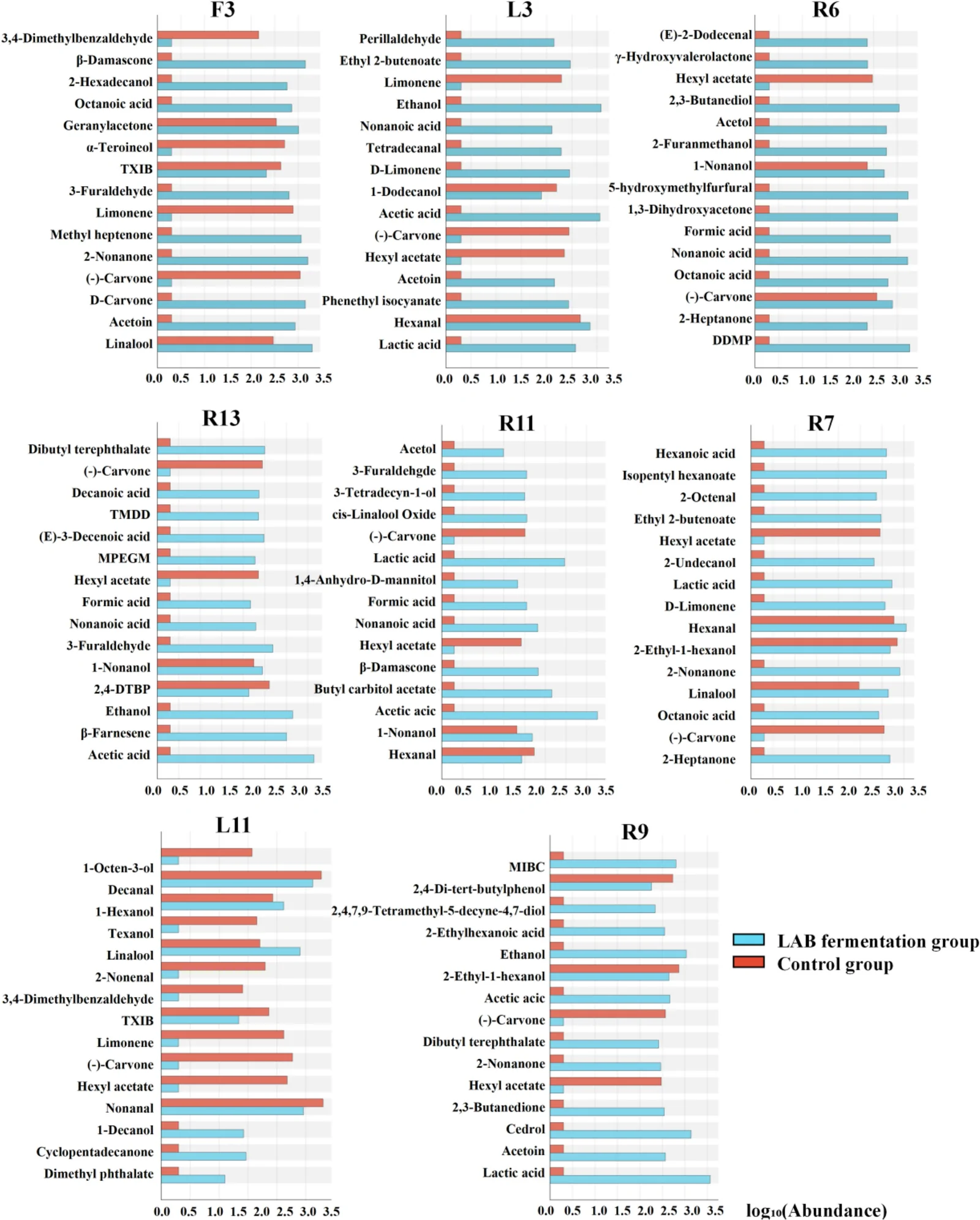
Pairwise comparisons of volatile metabolites between the unfermented and each fermented group (Con, L3, R11, L11, R7, R9, R6, R13, and F3). The top 15 volatile compounds exhibiting the most significant differences (based on *t*-test, *p < 0.05*) are shown. The X-axis represents the log10-transformed relative abundance.

Metabolite profiles significantly vary among bacterial strains due to differences in metabolic enzyme systems, directly affecting the flavor of fermented products. Fermentation with *L. plantarum* L3 resulted in the greatest reduction in carvone content in Dangshan Su pears; meanwhile, acetic acid, lactic acid, ethyl 2-butenoate, and d-limonene showed the most significant increases (Fig. 5). Ethyl 2-butenoate is a short-chain fatty acid ethyl ester, featuring fruity, rum-like, and sweet notes. In contrast, d-limonene is a monocyclic terpene characterized by a typical citrus aroma. Consistent with our findings, Ma et al. (2025) reported that *L. plantarum* fermentation enhanced terpenoid release in fruit and vegetable matrices. These metabolic changes lean toward a flavor profile defined by sourness, citrus, and fruity sweetness in the fermented pear, while reducing its original herbal or minty notes. Following fermentation with L. *paracasei* F3, the contents of nonanal, decanal, and carvone in Dangshan Su pears significantly decreased, whereas those of linalool and 2-nonanone showed the greatest increase among all volatile metabolites.

Following fermentation with *L. fermentum* R11, the strain exhibited an outstanding capacity to produce acid compounds. The content of volatile flavor compounds increased most significantly, with acetic acid as the primary contributor at 157.37 μg/L, followed by lactic acid and octanoic acid. Notably, the contents of d-mannitol and sorbitol increased significantly to 14.31 and 7.05 μg/L, respectively. This suggests that the strain may possess a unique polyol metabolic capability that enhances the product's sweetness and mouthfeel and confers functional properties (Ortiz et al., 2013). This is consistent with the findings of Tlais et al. (2024), who reported increased mannitol levels in fermented apple-pear juice. These metabolic changes favor the development of a flavor profile characterized by refreshing sourness, fatty notes, and sweetness in fermented Dangshan Su pears, potentially yielding a more balanced, enriched flavor. Sugar-lowering effects, coupled with mannitol accumulation, positively modulate the gut microbiota and intestinal integrity (Tlais et al., 2024). Therefore, our findings support the emerging evidence suggesting that LAB-fermented fruit products with reduced sugar and enhanced mannitol may serve as healthier alternatives to traditional fruit juices.

Fermentation of Dangshan Su pears with *Lc. mesenteroides* R6 and *Lc. lactis* R13 has a distinct metabolic profile. R6 fermentation significantly increased acetoin (26.52 μg/L), 2,3-butanediol, and linalool, contributing buttery, creamy, and floral notes, consistent with previous findings showing that *Leuconostoc* species produce diacetyl and acetoin via citrate metabolism under acidic conditions (Hemme & Foucaud-Scheunemann, 2004). Specifically, R13 enhanced (*E*)-β-farnesene (10.15 μg/L), imparting a green, woody, or fruity aroma, and increased the levels of 3-furaldehyde, 1-hexanol, and a cyclohexadienone derivative, suggesting strain-specific formation of furanoids and unsaturated ketones. These strain-specific metabolic traits are consistent with previous reports demonstrating that the *Leuconostoc* strains selectively modulate volatile compound profiles during fruit fermentation (Di Cagno et al., 2016). Both strains reduced several aldehydes (decanal and 2-nonenal) and limonene, decreasing green and citrus off-notes. However, unlike R6, R13 decreased the levels of (−)-carvone, hexyl acetate, and 2,4-di-tert-butylphenol. These results indicate that *Lc. mesenteroides* R6 favors diol and monoterpenoid enhancement, while *Lc. lactis* R13 promotes farnesene and specific alcohol accumulation.

Three *Lactococcus* strains fermented Dangshan Su pears with distinct metabolic patterns. *L. cremoris* R7 uniquely increased 2-nonanone (6.63 μg/L) and 2-heptanone, both methyl ketones known to impart blue-cheese and fruity aromas. Additionally, R7 increased the hexanal content, potentially contributing to the fresh green notes. *L. lactis* subsp. *lactis* R9 specifically increased the d-carvone and linalool levels, suggesting strain-specific terpenoid conversion capabilities, possibly via redox enzymes. Notably, *L. lactis* L11 produced the highest acetoin and 2,3-butanedione (buttery/diacetyl note), along with cedrol (10.26 μg/L) — a woody sesquiterpene alcohol unique to this strain (Smit et al., 2005). All three strains consistently reduced the levels of (−)-carvone, 2,4-di-tert-butylphenol, hexyl acetate, limonene, and 2-nonenal, indicating common metabolic pathways for the reduction of green, citrus, and off-flavor compounds. However, only L11 substantially increased the levels of acetic acid (27.49 μg/L) and octanoic acid, suggesting stronger lipolytic activity. These results demonstrate that strain-level selection within *Lactococcus* critically determines the final flavor profile of fermented pear products.

### 3.6 Sensory evaluation

**Fig. 6:**
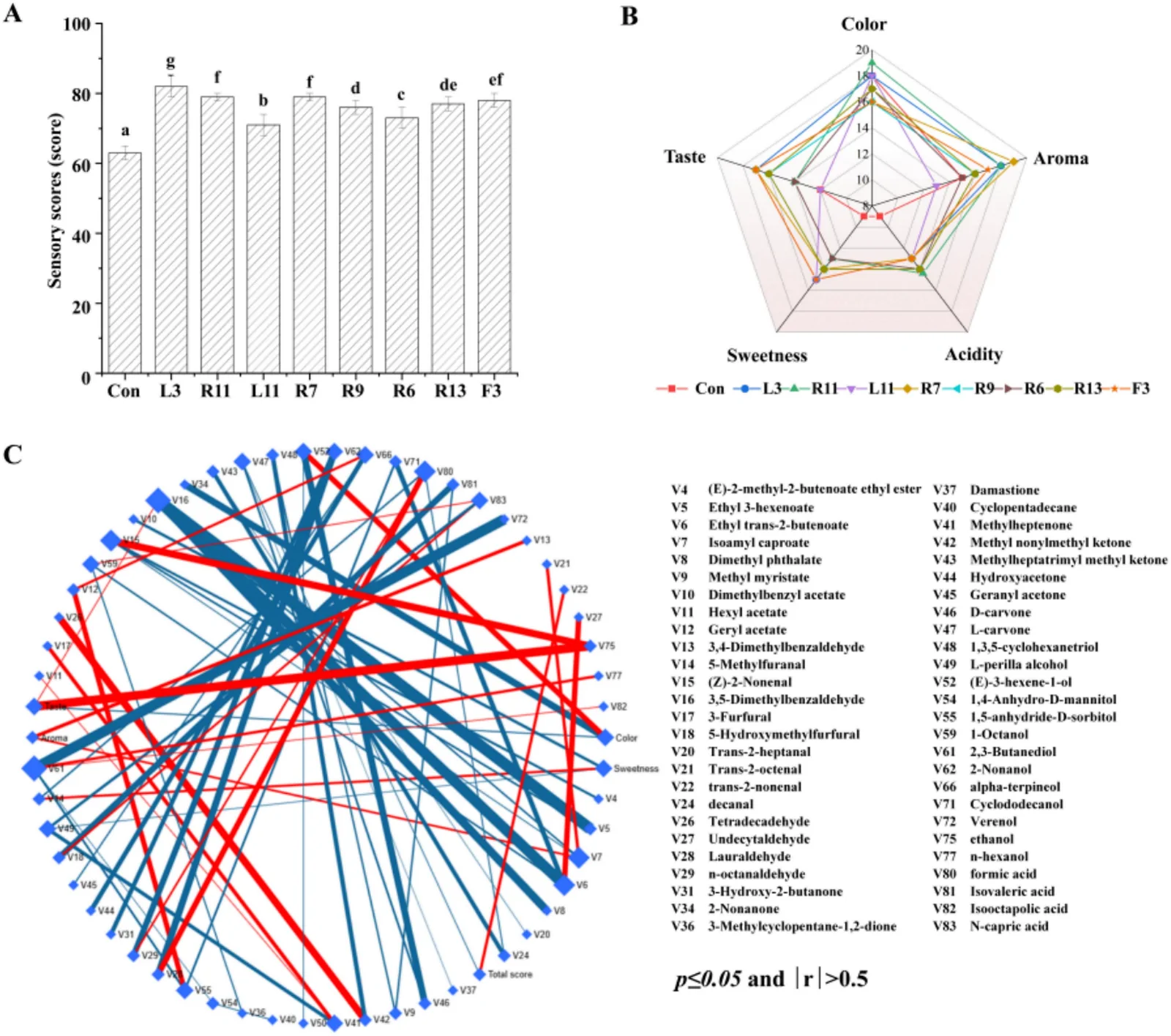
Sensory evaluation and correlation network analysis of unfermented and LAB-fermented Dangshan Su pears (Con, L3, R11, L11, R7, R9, R6, R13, and F3). (A) Total sensory scores across different groups (*n* = 12 panelists). Bars labeled with different lowercase letters indicate significant differences (*p < 0.05*) based on ANOVA followed by LSD post-hoc test. (B) Radar chart illustrating the sensory attribute profiles (color, aroma, acidity, sweetness, and taste). Each axis represents the score of a specific attribute. (C) Correlation network linking volatile compounds to sensory attributes (Aroma, Taste, Sweetness, Color, and Total score). Only significant correlations with an absolute correlation coefficient |r| > 0.5 and *p ≤ 0.05* are displayed.

Fig. 6 shows the results of the sensory evaluation of Dangshan Su pears before and after fermentation. The total sensory scores of samples fermented with the eight LAB strains were significantly higher than those of the unfermented control, indicating that LAB fermentation effectively enhanced the overall sensory quality. Among the strains, L3 exhibited the highest total score (81.50), followed by R11 and R7. All fermented groups scored significantly higher than the control for both sourness and sweetness, suggesting that organic acids and carbohydrate-derived metabolites enhanced the sweet–sour balance. L11-fermented samples achieved the highest color score, suggesting that L11 is the most effective for color protection and suitable for products requiring high color stability. Although R7 had the highest aroma score, likely due to the accumulation of fruity ester compounds, L3 showed no significant difference from R7 in taste, and both strains obtained the highest taste scores.

To further explore the chemical basis of these sensory differences, a network analysis was conducted to link the volatile profiles to the sensory attributes (Fig. 6C). A strong positive correlation between Taste score and ethanol (V75) suggests ethanol enhances mouthfeel and serves as a solvent for flavor release. 3,4-Dimethylbenzaldehyde (V13) and isoamyl caproate (V7) were positively correlated with Aroma score, implying their involvement in enhancing the fruity and nutty aromatic complexity. 5-Methylfuranal (V14) exhibited a positive correlation with Sweetness score, suggesting that the caramel-like and roasted notes of this furan derivative may enhance the perception of sweetness. In contrast, Dimethylbenzyl acetate (V10) displayed a negative correlation with Sweetness, suggesting that high concentrations of this compound might mask the sweet taste or introduce conflicting sensory cues. Moreover, the Color score showed a strong positive correlation with (E)-3-hexene-1-ol (V52), indicating its potential role in pigment retention or color stability. Total score was positively associated with trans-2-nonenal (V22), suggesting it acts as a flavor enhancer in this specific matrix. Overall, the differences in sensory scores among the strains were primarily attributable to variations in metabolic profiles, highlighting L3 and R11 as promising starter cultures for improving the sensory qualities of fermented Dangshan Su pears.

### 3.7 Non-volatile metabolites of Dangshan Su pear after fermentation

**Fig. 7:**
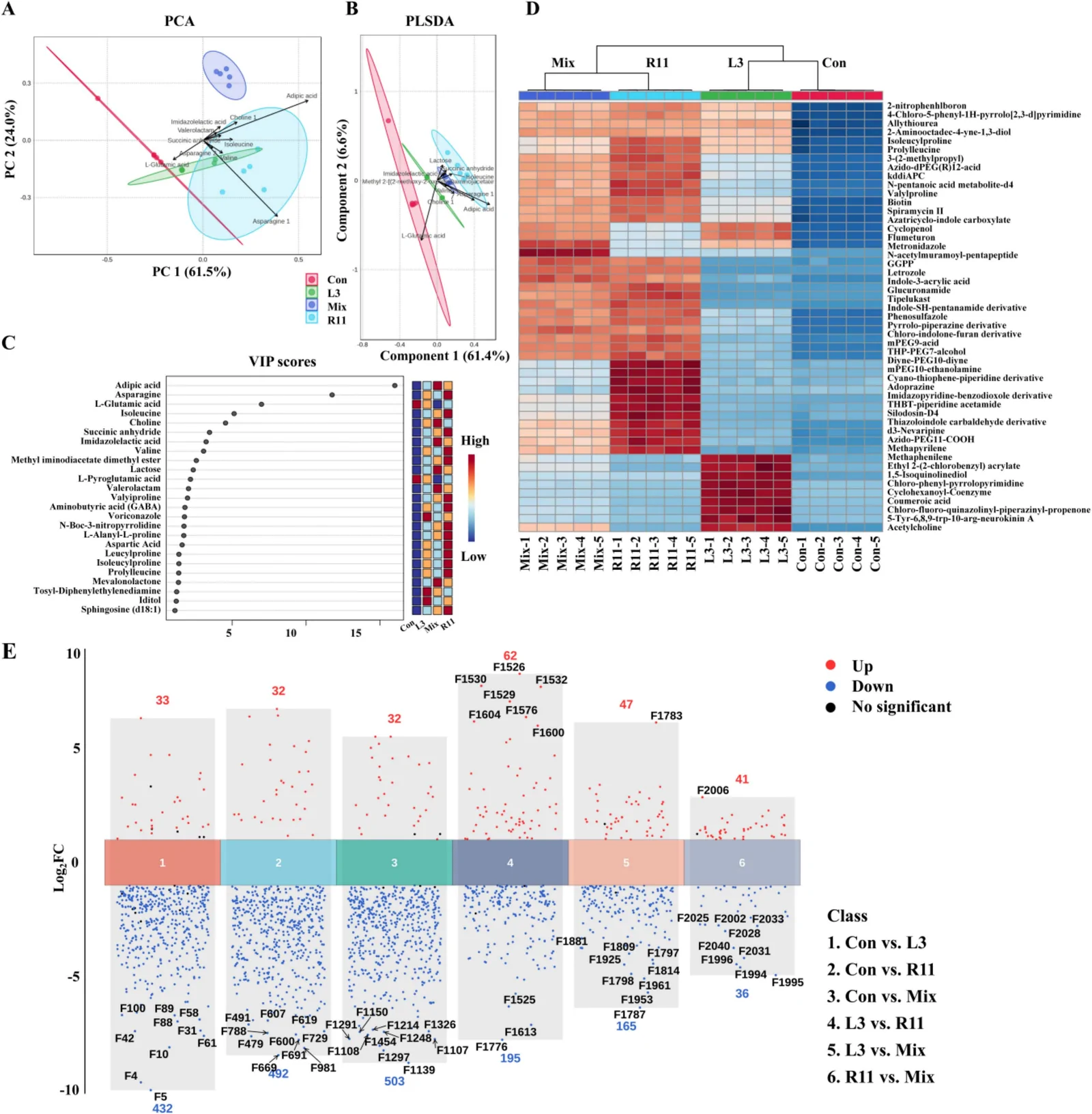
Non-volatile compound profiles of Dangshan Su pears fermented by *L. plantarum*, *L. fermentum*, and their mixed culture (Con: unfermented control; L3: *L. plantarum* L3-fermented group; R11: *L. fermentum* R11-fermented group; Mix: 1:1 co-culture of L3 and R11). (A) PCA score plot. (B) PLS-DA score plot. (C) VIP scores from the PLS-DA model. (D) Heatmap of differential non-volatile compounds (top 50, *p < 0.05*, *n* = 5). The color scale represents the relative abundance (red indicates high, blue indicates low). (E) Volcano plots for pairwise comparisons of non-volatile metabolites among the four groups. Differential metabolites were selected based on VIP > 1.0, |log2(FC)| > 1, and adjusted p (q) < 0.05. (For interpretation of the references to color in this figure legend, the reader is referred to the web version of this article.)

Based on the above findings, *L. plantarum* L3, *L. fermentum* R11, and their co-cultures were selected to ferment Dangshan Su pears. Non-targeted metabolomics was employed to determine the effects of fermentation on the profiles of non-volatile compounds. A total of 594 metabolites were detected across the four groups. The principal component analysis (PCA) score plot (Fig. 7A) showed a clear separation between the unfermented and fermented groups, with the PCA model yielding an R^2^X of 0.870. The PLS-DA model demonstrated excellent explanatory and predictive performance (R^2^X = 0.870, R^2^Y = 0.994, Q^2^ = 0.987), and permutation testing (200 iterations) confirmed model robustness with a Q^2^ intercept of −0.425 and an R^2^ intercept of 0.117, ruling out overfitting. The unfermented group (Con) was distinctly separated from all fermented groups along the PC1 axis, indicating that fermentation induced global metabolic remodeling. Furthermore, the *L. plantarum* L3-fermented (L3), *L. fermentum* R11-fermented (R11), and mixed fermentation (Mix) groups were partially distinguished, suggesting that different strains and their combinations exert specific regulatory effects on metabolic profiles.

VIP analysis identified the top 25 non-volatile compounds that contributed to group discrimination. The heatmap/clustering analysis revealed distinct accumulation patterns (Fig. 7C and D). Adipic acid displayed the highest VIP value among all identified metabolites, establishing it as the most important discriminatory compound between unfermented and fermented samples, and among the three fermentation modes. Moreover, mixed fermentation yielded the highest adipic acid concentration, suggesting an additive effect on adipic acid biosynthesis compared with single-strain fermentation. Asparagine was also identified as an important discriminatory metabolite among the four groups, reaching peak concentration in Dangshan Su pears fermented by *L. fermentum* R11. As a free amino acid that contributes to sweet and umami flavors, the enrichment of asparagine improves the taste profile of fermented fruits (Zhao et al., 2016). In contrast, L-glutamic acid and pyroglutamic acid levels were higher in the unfermented group than in all fermented groups, indicating that fermentation consumed these amino acids or converted them into derivatives.

All other 23 compounds—including isoleucine, valine, proline, GABA, choline, and various dipeptides (leucylproline, isoleucylproline, and prolylleucine)—significantly increased post-fermentation. The notable increases in branched-chain amino acids, proline, and GABA reflect interconnected metabolic processes. As fermentable sugars are progressively consumed via glycolysis, LAB strains shift their metabolism toward amino acid catabolism and biosynthesis to sustain energy demands and redox balance (Gänzle, 2015). The increase in valine and isoleucine likely reflects enhanced branched-chain amino acid biosynthesis, and these amino acids serve as precursors for Strecker aldehydes that contribute fruity and malty notes (Smit et al., 2005). Proline functions as an osmoprotectant under acidic conditions while also contributing sweet taste (Zhao et al., 2016). Most notably, GABA exhibited the most pronounced upregulation (4.62-fold after R11 fermentation). The elevated GABA levels observed in this study are consistent with previous findings in *L. plantarum*-fermented wolfberry juice, in which GABA accumulation was a characteristic secondary metabolite contributing to taste improvement (Zhang et al., 2022). It is synthesized via α-decarboxylation of L-glutamate by glutamate decarboxylase (GAD), and its accumulation serves as a protective mechanism against acid stress (Feehily & Karatzas, 2013). The particularly high GABA yield of *L. fermentum* R11 may reflect its superior GAD activity under acidic pear conditions. GABA is recognized for its hypotensive, anxiolytic, and neuroprotective activities (Diana et al., 2014).

Other upregulated metabolites included choline and various dipeptides (leucylproline, isoleucylproline, and prolylleucine). As a quaternary ammonium compound, choline was also significantly elevated across all fermented groups. It can be synthesized by LAB or released from pear phospholipids via microbial phospholipases (Kleerebezem et al., 2003). As an essential nutrient, choline serves as a precursor for acetylcholine and phosphatidylcholine, and adequate intake has been linked to improved cognitive function, liver health, and cardiovascular protection (Zeisel & da Costa, 2009). The increase in dipeptides reflects the proteolytic activity of LAB via cell-envelope proteinases and intracellular peptidases (Savijoki et al., 2006), with distinct profiles between L3 and R11 arising from differences in enzyme specificities. Notably, certain dipeptides exhibit ACE-inhibitory and antioxidant activities, while leucylproline and prolylleucine contribute to kokumi taste, potentially explaining the improved mouthfeel of R11-fermented samples (Rhyu & Kim, 2011). Collectively, dipeptide enrichment enhances both flavor complexity and functional value.

Metabolite set enrichment analysis (Fig. S3) revealed that the enriched pathways were primarily associated with amino acid metabolism (e.g., branched-chain amino acid degradation, glutamate metabolism), stress responses (glutathione metabolism), and ammonia recycling. These findings provide systematic validation of the interconnected metabolic processes, including the enhanced antioxidant defense system and the GABA shunt serving as an acid stress adaptation mechanism. Collectively, fermentation with *L. plantarum* L3 and *L. fermentum* R11, either singly or in combination, effectively enriched a wide range of non-volatile bioactive and flavor-related compounds in Dangshan Su pears, with strain-specific and synergistic metabolic outcomes that can facilitate targeted flavor modulation and nutritional improvement. While the present metabolomics approach successfully revealed extensive metabolic remodeling induced by the selected strains, the enzymatic drivers of phenolic conversion and GABA accumulation require future proteomic or enzymatic assays.

Volcano plot analysis revealed distinct alterations in the non-volatile metabolites of Dangshan Su pears among the unfermented, *L. plantarum* L3-fermented, *L. fermentum* R11-fermented, and mixed fermentation groups (Fig. 7E). Compared to the unfermented group, all fermented samples exhibited a massive upregulation of primary pear metabolites, accompanied by moderate downregulation of specific metabolites. Among them, mixed fermentation showed the strongest catabolic effect, indicating synergistic interactions between the two strains. Among the identified non-volatile metabolites, p-coumaric acid (p-CA) showed the most significant increase following fermentation. This accumulation can be attributed to the enzymatic degradation of pear cell wall polysaccharides during LAB fermentation, which facilitates the release of cell wall-bound phenolics, including p-coumaric acid (Isas et al., 2020). p-CA is a hydroxycinnamic acid widely distributed in fruits, vegetables, and cereals with biological activities that contribute to its protective roles against various oxidative stress-related diseases, including cardiovascular diseases, diabetes, and neuroinflammation (Roychoudhury et al., 2021). Strain-specific differences between *L. plantarum* L3 and *L. fermentum* R11 fermentations were reflected by the significant upregulation of N-feruloyl octopamine [F1526] and downregulation of phenylalanine [F1776]. Moreover, compared with *L. plantarum* fermentation, mixed fermentation significantly upregulated 5-guanidino-2-oxopentanoic acid [F1797] and indole-3-acrylic acid [F1809] and downregulated L-glutamyl-L-leucine [F1783]. In contrast, slight differences (e.g., coumaric acid [F1995]) were observed between *L. fermentum* R11 and mixed fermentations, suggesting that *L. fermentum* R11 is the dominant influencer in shaping the metabolic phenotype during mixed fermentation.

## 4 Conclusion

In this study, we evaluated the fermentation performance of eight lactic acid bacterial strains in the Dangshan Su pear matrix. Among the strains tested, *L. plantarum* L3 and *L. fermentum* R11 were identified as promising starters. L3 excelled in acid production and antioxidant enhancement, while R11 contributed distinctive volatile profiles and functional metabolites. Their co-culture further demonstrated enhanced metabolic activity, validating multi-strain formulations as an effective strategy for modulating flavor complexity and nutritional quality. Importantly, the strain-dependent metabolic variations observed underscore the necessity of targeted starter selection for this substrate. Furthermore, the integrated physicochemical, sensory, and metabolomic framework is transferable to other fruit substrates. Moreover, the mild fermentation conditions and food-grade nature of the process ensure seamless integration into existing production lines, offering a viable strategy for upgrading the industrial value of this widely cultivated pear variety. Future efforts should focus on elucidating strain-specific regulatory networks, assessing long-term storage stability, and evaluating consumer sensory acceptance to support pilot-scale translation.

## Declaration of generative AI use

No generative AI or AI-assisted tools were used for data analysis, figure generation, or the drawing of scientific conclusions.

## CRediT authorship contribution statement

**Jiayu Gu:** Writing – original draft, Investigation, Data curation. **Qian Zhu:** Writing – review & editing, Conceptualization. **Jingjing Du:** Writing – review & editing, Methodology. **Jiagang Guo:** Conceptualization. **Yuhan Wu:** Supervision, Resources, Methodology. **Shuo Wang:** Writing – review & editing, Methodology. **Jian Jiang:** Supervision, Conceptualization. **Song Yang:** Supervision, Resources, Project administration.

## Declaration of competing interest

The authors declare that they have no known competing financial interests or personal relationships that could have appeared to influence the work reported in this paper.

## Data Availability

Data will be made available on request.
