# Supplementary material for: Physicochemical, nutritional, and flavor attributes of Dangshan Su pear fermented with various lactic acid bacteria
Source: Food Chem X. 2026 Aug 27;39:104338. doi: 10.1016/j.fochx.2026.104338 (PMC13571294; doi:10.1016/j.fochx.2026.104338)
Supplement: Supplementary file 1 [file mmc1.docx]

**Tables**

**Table S1** The strains used in fermentation of Dangshan Su pear

| Species | Strain | Origin | GenBank accession number | Hemolytic activity |
| --- | --- | --- | --- | --- |
| *Lactiplantibacillus plantarum* | L3 | Pickle | PZ770652 | Negative |
| *Limosilactobacillus fermentum* | R11 | Bamboo shoot | PZ770653 | Negative |
| *Lactococcus lactis* | L11 | Bamboo shoot | PZ770659 | Negative |
| *Lactococcus cremoris* | R7 | Bamboo shoot | PZ770655 | Negative |
| *Lactococcus lactis subsp. lactis* | R9 | Bamboo shoot shell | PZ770656 | Negative |
| *Leuconostoc mesenteroides* | R6 | Bamboo shoot shell | PZ770654 | Negative |
| *Leuconostoc lactis* | R13 | Bamboo shoot shell | PZ770657 | Negative |
| *Lacticaseibacillus paracasei* | F3 | Bamboo shoot shell | PZ770658 | Negative |

**Table S2** Organic acid standard curve

| Organic acid | Retention time (min) | Calibration equation | R^2^ |
| --- | --- | --- | --- |
| Tartaric acid | 9.85 | y = 1120x - 3435.6 | 0.9919 |
| Oxalic acid | 4.53 | y = 10018x - 149794 | 0.9993 |
| Citric acid | 4.89 | y = 1670.9x - 5949.7 | 0.9985 |
| Malic acid | 5.73 | y = 1753.8x - 31357 | 0.9911 |
| Lactic acid | 7.54 | y = 632.32x - 15407 | 0.9913 |
| Acetic acid | 7.11 | y = 714911x - 6351.5 | 0.9967 |

**Table S3** Sensory evaluation criteria for fermented Dangshan Su pear

| Item (Score) | Description | Score |
| --- | --- | --- |
| Color (20) | Bright color, presenting the natural white or light yellow | 15 ~ 20 |
|  | Dull color, presenting light gray or light brown | 9 ~ 14 |
|  | Dark color, presenting gray or yellowish brown | <9 |
| Acidity (20) | Moderate acidity, refreshing | 15 ~ 20 |
|  | Slightly excessive or mild acidity | 9 ~ 14 |
|  | Overly acidic or insufficient acidity | <9 |
| Sweetness (20) | Moderate sweetness, well-balanced | 15 ~ 20 |
|  | Slightly excessive or mild sweetness, slightly unbalanced | 9 ~ 14 |
|  | Insufficient or excessive sweetness, severely unbalanced | <9 |
| Aroma (20) | Rich aroma, well-coordinated with pear and fermented notes | 15 ~ 20 |
|  | Slightly unbalanced aroma, insufficient or excessive fermented notes | 9 ~ 14 |
|  | Pungent or off-flavor | <9 |
| Taste (20) | Palatable, well-balanced, and pleasant | 15 ~ 20 |
|  | Slightly unbalanced taste | 9 ~ 14 |
|  | Bland, abnormal, or uncoordinated taste | <9 |

**Figure S1** Pearson correlation matrix of bioactive compounds and functional activities. The numbers represent Pearson correlation coefficients (r). The color scale indicates the direction and strength of the correlations (red: positive, blue: negative). Asterisks indicate statistical significance (* *p < 0.05*, ** *p < 0.01*).

**Figure S2** Permutation test (n = 200) for the PLS-DA model comparing the four groups (Con, L3, R11, and Mix). The intercepts of the regression lines were R² = 0.117 and Q² = -0.425. The Q² intercept below zero confirms that the model was not overfitted and that the observed group discrimination was statistically reliable.

**Figure S3** Metabolite set enrichment analysis (MSEA) of the top 25 enriched metabolic pathways in fermented Dangshan Su pears. The X-axis indicates significance (−log_10_p). Circle size represents the enrichment ratio, and color intensity corresponds to the p-value (warmer colors indicate greater significance).
